# Alloy as a Filling Material

**Published:** 1899-03

**Authors:** W. H. Chilson

**Affiliations:** Appleton, Wis.


ARTICLE X.

### ALLOY AS A FILLING MATERIAL.

W. H. CHILSON, D. D. S., APPLETON, WIS.

**Read before the Wisconsin State Dental Society.**

Conservatism in dentistry, if it means anything, means that all teeth can reasonably be retained through life, and a very substantial progress in the art of censervatism can be noted by the older class of practicing dentists. Hope lies in this fact, and to this end we cast about to discover any and all means that may aid us in our purpose. The public are more critical exacting and appreciative in these later days, and through our teachings are enabled to form an opinion that will stand the test. They appreciate the alloy filling, because it can be made in one-fourth of the time with less pain and expense than a gold filling, and there are cases where an alloy filling will serve the purpose as well as gold filling. These facts should never be overlooked, because you render a service that saves time, money and gives comfort, all of which if well performed gives satisfaction to the most exacting of our patients. To-day we are rated as failures just in proportion to our losses of fillings that we make for treatment in conserving teeth.

In our early practice the mechanical part of dentistry was thought to be, by the then discriminating public, the highest attainment of the dental art and for the best results people would make pilgrimages of long distances to reach the experts in that line, for in those days artificial dentures were used more in proportion to other dental operations than they are to-day, and the high appreciation of them in former days kept up their price, so that the best efforts of our profession were directed along that line. An opinion was very prevalent at that time that sooner or later everybody would be obliged to wear artificial teeth, because operative dentistry did not then fulfill the expectations of our patients, and the many failures of alloy fillings were excused on the ground that they were only a temporary expedient at best, and charges for those services were made accordingly. Happily for humanity these two articles have changed places in the minds of a discriminating public, and the alloy filling has taken the place its merit entitled it to, and the other, I am sorry to admit, is in the hands of the six-dollar-a-set man so much that their usefulness is greatly impaired.

As the years have come and gone, among the additional requirements and responsibilities impressed on one, is the larger range of usefulness of dentistry to the great multitude of humanity whose means have been and always will be meager, yet whose dental requirements are even greater than those whose means are adequate for their comfort.

We should measure our success by the amount of good that we can do our fellow men, and this thought brings me more closely to my subject.

It is not my purpose to enter into the relative merits of the different filling materials, but rather to call your attention more closely to what may be relied upon in the construction of an alloy filling.

Original investigations in alloys and amalgams by a number of our leading professional lights have resulted in much good information of which we can avail ourselves. Among their deductions are the following facts: That amalgam is susceptible of good edge strength, capable of sustaining the necessary occlusal pressure, color and polish, and the more carefully these properties are brought out, the less shrinkage there will be.

To make a good alloy filling as much care must be used in preparation as for a gold filling, and three principles must be kept in the mind of the operator in order that he may do his best in every case. First, the cavity of decay may be prepared in the manner dictated by the latest and most approved system of dental technics; second, the alloy should be so skillfully mixed that all of its parts shall receive a like quantity of mercury and force applied at the same time so that the hardening shall be simultaneous; third, careful and skillful manipulation must be exercised to obtain the best results. Amalgams or alloys that have soft spots and an uneven surface are due to the lack of careful attention in mixing, and sometimes due to introducing the mass after the hardening has set in. Another cause for soft spots in the alloy is too much pressure under the instrument; the tendency is that by overpressure the precious metals flow away with free mercury to the edge or cavity walls; this process leaves the filling to harden disproportionately, and as a result we have a surface soft in spots and edge strength impaired, a surface that will not admit of dressing and polishing in a satisfactory manner. The shrinkage in such case is very marked and by proper care can be largely overcome. After the filling is placed attention should be directed to the occlusal pressure, and if this be not attended to faithfully, fracture and displacement will result and render our best efforts unavailing. My own method for overcoming these difficulties I fully believe reduces them to a minimum. I repeat then that the basis for the best results is the skillfulness with which the cavity is prepared for the retention of the filling. To illustrate, we will suppose a case in hand to be a distal cavity in a superior second bicuspid, nothing unusual, just an everyday affair. We should first place the rubber dam in position, then with chisels remove all overhanging enamel until supported by sound dentine, then remove decay and outline the cavity as follows: The base or gingival wall should be made perfectly flat and should extend below the free margin of the gum to render it self-cleansing, the axial walls should be extended to the lingual and buccal surfaces and should be perpendicular to the base; then with fissure-bur cut out fissure on the occlusal surface and make a dove-tailed extension for retention, and then a reasonable undercut may be made all around the margins, so that if the cavity were an ideal one the filling would be keyed in and would take the appearance of a keystone in an arch. I believe too much stress can not be laid on the preparation of the alloy, because its density, color, edge strength and shrinkage depend altogether upon the manner in which it is treated in its formative stages. After discarding the mortar and pestle for mixing, the chamois skin for expelling excess of mercury, sodium and alcohol for washing to improve color, my method is simply this: Place in the palm of the hand a sufficient quantity of alloy, then add mercury from time to time, and rub both together with the index finger until a stiff paste producing a creaking noise when rolled under pressure is produced. The shape of the mass at this stage will be oval and the ends will be softer than the center, due to the fact that the mercury has been forced to the ends; so the mass is pressed longitudinally, which produces an equal density throughout, and is then cut into small pellets with a sharp instrument. Thus far we have met the condition in the preparation of the cavity and alloy, and although we proceed confident of good results, much still depends upon the manner in which we introduce the alloy into the cavity, for it requires careful manipulation to produce the required results.

Be careful, then, to place in the cavity small pieces of amalgam pressed snugly against the cavity walls by small, flat, smooth instruments, which should be repeated until a sufficient amount has been introduced. A final finish should also receive our best efforts, and what we should strive to prevent at this stage is burnishing, because it brings the mercury to the surface with the objections which we have before stated. Use sharp, thin cutting instruments to contour and finish, and at a second sitting after the amalgam is thoroughly hardened polish in the usual manner with strips and disks, and you will find that it is susceptible of a very high polish.—*Dental Century.*

